# Chloroplast genome of *Trapa bispinosa* Roxb. (Trapa, Lythraceae)

**DOI:** 10.1080/23802359.2020.1866457

**Published:** 2021-02-05

**Authors:** Lin Lin, Jie Wang, Yu-Xue Zhao, Li Ma, Cui-Hua Gu, Zhi-Qiang Wu

**Affiliations:** a School of Landscape and Architecture, Zhejiang A & F University, Hangzhou, China; b Zhejiang Provincial Key Laboratory of Germplasm Innovation and Utilization for Garden Plants, Zhejiang A & F University, Hangzhou, China; c Key Laboratory of National Forestry and Grassland Administration on Germplasm Innovation and Utilization for Southern Garden Plants, Zhejiang A & F University, Hangzhou, China; d College of Forestry and Biotechnology, Zhejiang A & F University, Hangzhou, China; e Shenzhen Branch, Guangdong Laboratory for Lingnan Modern Agriculture, Genome Analysis Laboratory of the Ministry of Agriculture, Agricultural Genomics Institute at Shenzhen, Chinese Academy of Agricultural Sciences, Shenzhen, China

**Keywords:** *Trapa bispinosa*, complete chloroplast genome sequence, phylogenetic analysis

## Abstract

*Trapa bispinosa* Roxb. is an annual aquatic herb with great significance of medicinal, edible and economic value. Here, we reported the complete chloroplast genome sequence of *Trapa bispinosa* and conducted preliminary investigation of its phylogenetic relationship with other related species. As the result showed, the whole chloroplast genome size was 155,556 bp consisting of four adjoining regions, i.e., a large/small single copy (LSC, 88,506 bp/SSC, 18,274 bp) region and two inverted repeat (IRs, 24,388 bp) regions. Among 112 identified unique genes were 78 protein coding genes, 30 transfer RNA (tRNA) genes, and four ribosomal RNA (rRNA) genes. *Trapa* spp. were precisely clustered as a monophyly, and simultaneously, the closest relation between *Trapa bispinosa* and *Trapa natans* were strongly supported in the maximum likelihood analysis.

*Trapa bispinosa* Roxb. (water chestnut) is an annual aquatic herb used to belong to Trapaceae, and currently is included in Lythraceae, which is named for its mature fruit shell with two longer and slightly curved sharp corners (Chen et al. 2007). The species mainly grows in shallow waters such as lakes and ponds (Gao et al. 2014). It has been widely cultivated around the world for its possession of great medicinal effects, plus economic, edible, and ecological functions (Adkar et al. 2014). Herein, we reported the complete chloroplast genome of *Trapa bispinosa* and analyzed its phylogenetic position within Lythraceae, aiming to provide genome information for its further research, development, and application.

We sampled fresh leaves of *Trapa bispinosa* from the cultivation pond of Zhejiang A&F University, Hangzhou, Zhejiang province, China (30°13′48″N, 119°43′12″E), and then stored them at Herbarium of Zhejiang A & F University with code ZAFU1907211 as the specimen. Genomic DNA was extracted following Doyle (1987) and Yang et al. (2014). The sequencing library based on total DNA was established with the average insert size of 350 bp, and 6.71GB raw data with 150 bp paired-end reads generated by the Illumina HiSeq2500 platform (Shenzhen, China) and further filtered via Trimmomatic v0.3 (Bolger et al. 2014). De novo assembly was accomplished by CLC v9.11 (Nicolas et al. 2017). Taken *Trapa maximowiczi* (Xue et al. 2017) chloroplast genome as the reference, contigs alignment was accomplished under the BLAST (Johnson et al. 2008). The online tool GB2sequin (https://chlorobox.mpimp-golm.mpg.de/GenBank2Sequin.html) (Lehwark and Greiner 2019) was utilized to annotate the genome. MsatCommander v0.8.2.0 (Faircloth 2008) was utilized for the identification of simple sequence repeats (SSRs). Finally, information on the sequence was submitted to GenBank (Accession number: MW065552).

Four adjacent parts formed the 155,556 bp circular chloroplast genome, namely, a large single copy (LSC, 88,506 bp) region, a small single copy (SSC, 18,274 bp) region, and a pair of inverted repeat (IRs, 24,388 bp) regions. Overall GC content reached 36.41%, additionally, 34.19% for LSC, 42.77% for IR, and 30.18% for SSC. There were in total 112 unique genes consisting of 78 protein coding genes, 30 transfer RNA (tRNA) genes and four ribosomal RNA (rRNA) genes, among which 14 (8 protein coding genes and four tRNA genes) contained one intron, besides, three (*rps12*, *clpP*, and *ycf3*) contained two introns. 332 SSRs were identified, including 198 Mono-nucletide, 55 di-nucletide, 66 tri-nucletide, 11 tetra-nucletide, and one each for both penta-nucletide and hexa-nucleotide.

Another 21 related species plastid sequences (18 from Lythraceae plus three outgroups from Onagraceae) were obtained from GenBank to dig the phylogenetic relationship of *Trapa bispinosa*. Followed alignment completed by MAFFT v7 (Katoh et al. 2019) was the maximum likelihood (ML) analysis performed in IQ-Tree (Nguyen et al. 2015) with the best-fit model (GTR + G) through ModelFinder (Kalyaanamoorthy et al. 2017). Obviously, four *Trapa* species were exactly clustered as a monophyletic clade, among which *Trapa bispinosa* showed the closest relationship with *Trapa natans* (Figure 1).

**Figure 1.**
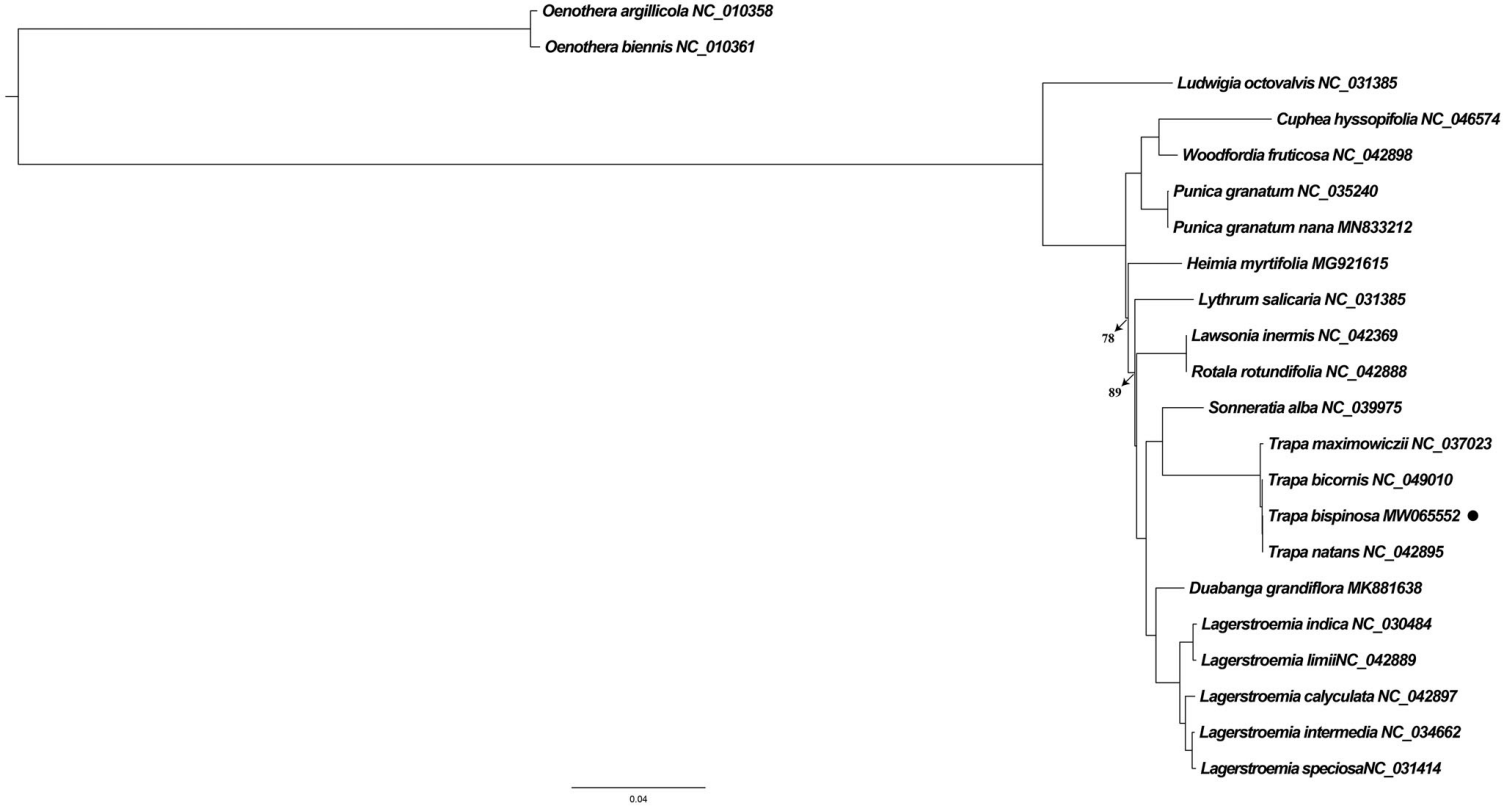
ML tree based on the whole chloroplast genome of 19 Lythraceae species with three Onagraceae species as outgroups. The numbers near nodes indicated the bootstrap value. 100% support value was not shown.

## Data Availability

The data that support the findings of this study are openly available in GenBank at https://www.ncbi.nlm.nih.gov/genbank/, reference number: MW065552.

## References

[CIT0001] Adkar P, Dongare A, Ambavade S, Bhaskar VH. 2014. *Trapa bispinosa* Roxb.: a review on nutritional and pharmacological aspects. Adv Pharmacol Sci. 2014:959830–959813. 24669216 10.1155/2014/959830 PMC3941599

[CIT0002] Bolger AM, Lohse M, Usadel B. 2014. Trimmomatic: a flexible trimmer for Illumina sequence data. Bioinformatics. 30(15):2114–2120. 24695404 10.1093/bioinformatics/btu170 PMC4103590

[CIT0003] Chen JR, Ding BY, Michele F. 2007. Flora of China. Vol. 53. Beijing, China: Beijing Science Press; p. 290–291.

[CIT0004] Doyle J. 1987. A rapid DNA isolation procedure for small quantities of fresh leaf tissue. Phytochem Bull. 19:11–15.

[CIT0005] Faircloth BC. 2008. msatcommander: detection of microsatellite repeat arrays and automated, locus-specific primer design. Mol Ecol Resour. 8(1):92–94. 21585724 10.1111/j.1471-8286.2007.01884.x

[CIT0006] Gao HM, Cai JW, Han WL, Huai H, Chen Y, Wei C. 2014. Comparison of starches isolated from three different *Trapa* species. Food Hydrocolloids. 37:174–181.

[CIT0007] Johnson M, Zaretskaya I, Raytselis Y, Merezhuk Y, McGinnis S, Madden TL. 2008. NCBI BLAST: a better web interface. Nucleic Acids Res. 36(Web Server issue):W5–W9. 18440982 10.1093/nar/gkn201 PMC2447716

[CIT0008] Kalyaanamoorthy S, Minh BQ, Wong TKF, von Haeseler A, Jermiin LS. 2017. ModelFinder: fast model selection for accurate phylogenetic estimates. Nat Methods. 14(6):587–589. 28481363 10.1038/nmeth.4285 PMC5453245

[CIT0009] Katoh K, Rozewicki J, Yamada KD. 2019. MAFFT online service: multiple sequence alignment, interactive sequence choice and visualization. Brief Bioinform. 20(4):1160–1166. 28968734 10.1093/bib/bbx108 PMC6781576

[CIT0010] Lehwark P, Greiner S. 2019. GB2sequin – a file converter preparing custom GenBank files for database submission. Genomics. 111(4):759–761. 29842948 10.1016/j.ygeno.2018.05.003

[CIT0011] Nguyen LT, Schmidt HA, von Haeseler A, Minh BQ. 2015. IQ-TREE: a fast and effective stochastic algorithm for estimating maximum-likelihood phylogenies. Mol Biol Evol. 32(1):268–274. 25371430 10.1093/molbev/msu300 PMC4271533

[CIT0012] Nicolas D, Patrick M, Guillaume S. 2017. NOVOPlasty: de Novo assembly of organelle genomes from whole genome data. Nucleic Acid Res. 45(4):e18. 28204566 10.1093/nar/gkw955 PMC5389512

[CIT0013] Xue Z, Xue J, Victorovna KM, Ma K. 2017. The complete chloroplast DNA sequence of *Trapa maximowiczii* Korsh. (Trapaceae), and comparative analysis with other *Myrtales* species. Aquat Bot. 143:54–62.

[CIT0014] Yang JB, Li DZ, Li HT. 2014. Highly effective sequencing whole chloroplast genomes of angiosperms by nine novel universal primer pairs. Mol Ecol Resour. 14(5):1024–1031. 24620934 10.1111/1755-0998.12251

